# Polyphenols Extracts from Oil Production Waste Products (OPWPs) Reduce Cell Viability and Exert Anti-Inflammatory Activity via PPARγ Induction in Colorectal Cancer Cells

**DOI:** 10.3390/antiox11040624

**Published:** 2022-03-24

**Authors:** Manuela Leo, Livio Muccillo, Laura Dugo, Roberta Bernini, Luca Santi, Lina Sabatino

**Affiliations:** 1 Department of Sciences and Technologies, University of Sannio, Via F. De Sanctis, 82100 Benevento, Italy; manuela.leo@unisannio.it (M.L.); livio.muccillo@unisannio.it (L.M.); 2 Department of Science and Technology for Humans and the Environment, University Campus Bio-Medico of Rome, Via Alvaro del Portillo 21, 00128 Roma, Italy; l.dugo@unicampus.it; 3 Department of Agriculture and Forest Sciences, University of Tuscia, Via San Camillo de Lellis snc, 01100 Viterbo, Italy; roberta.bernini@unitus.it (R.B.); luca.santi@unitus.it (L.S.)

**Keywords:** oil production waste products (OPWPs), polyphenols, hydroxytyrosol, apoptosis, cell cycle, inflammation, NF-κB, cytokines, PPARγ, cancer

## Abstract

Olive oil production is associated with the generation of oil production waste products (OPWPs) rich in water-soluble polyphenols that represent serious environmental problems. Yet OPWPs can offer new opportunities by exploiting their bioactive properties. In this study, we chemically characterized OPWPs polyphenolic extracts and investigated their biological activities in normal and colorectal cancer cells. Hydroxytyrosol (HTyr), the major constituent of these extracts, was used as the control. We show that both HTyr and the extracts affect cell viability by inducing apoptosis and cell cycle arrest. They downregulate inflammation by impairing NF-κB phosphorylation and expression of responsive cytokine genes, as *TNF-α* and *IL-8*, at both mRNA and protein levels, and prevent any further increase elicited by external challenges. Mechanistically, HTyr and the extracts activate PPARγ while hampering pro-inflammatory genes expression, acting as a specific agonist, likely through a trans-repression process. Altogether, OPWPs polyphenolic extracts show stronger effects than HTyr, conceivably due to additive or synergistic effects of all polyphenols contained. They display anti-inflammatory properties and these results may pave the way for improving OPWPs extraction and enrichment methods to reduce the environmental impact and support their use to ameliorate the inflammation associated with diseases and tumors.

## 1. Introduction

*Olea europaea* L. is one of the oldest trees in the Mediterranean area whose domestication dates back between 6000 and 8000 years ago [1] in the Anatolia peninsula in the area where Turkey and Syria are currently located. The long-lasting tradition of olive cultivation has its origin in social, cultural and religious reasons, considering that olives can be directly eaten or milled to produce olive oil with a relevant economic impact [1].

Olive oil is, in fact, one of the most representative constituents of the Mediterranean diet [2,3] and, not by chance, olive cultivation and olive oil productions are some of the most widespread agricultural and industrial activities in many countries of the Mediterranean Basin. The worldwide production of extra virgin olive oil is around 3,034,000 t, 67.8% of which is in Europe. Specifically, Spain (1,400,000 t), Italy (270,000 t), Greece (270,000 t) and Portugal (100,000 t) cover 99% of the European olive oil production; the remaining ~32% of worldwide production comes from North African countries and Turkey and South American countries, mainly Argentina and Paraguay [4].

This thriving industry is unfortunately associated with the production of a high quantity of waste, collectively named oil production waste products (OPWPs), consisting of oil-free olive pulp (solid) and olive mill wastewaters (liquid) from the olive washing process as well as from olive oil purification procedures. Both products raise severe environmental concerns due to the polyphenols contained, which are soluble compounds able to infiltrate and pollute the ground [5]. It is estimated that for every liter of extra virgin olive oil, 2.5 L of corresponding wastewaters are produced, for an annual world amount of ~8,000,000 t [6,7].

In the last decade, increasing interest has been devoted to find new procedures to dispose of wastewaters with the aim to remove or reduce these molecules. These techniques, in fact, usually tend, on one hand, to recover the bioactive molecules contained and, on the other hand, to re-utilize most of the remaining water in agriculture for irrigation or industrial purposes, meeting the principles of ecofriendly and circular economy for sustainable development [8,9,10,11,12]. The dangerous pollutants can also be recycled and transformed in either biofuels or biopolymers to be used for green packaging [13,14].

Polyphenols are natural compounds endowed with interesting biological properties such as antioxidant, anti-inflammatory, antiproliferative and hypoglycemic activities, and for these reasons their uses as nutraceuticals and additives in cosmetics and foodstuffs are increasing [15,16]. The most abundant compound in OPWPs is 2-(3,4-dihydroxyphenyl)ethanol (hydroxytyrosol, HTyr), which notoriously bears very high antioxidant potential [17]. OPWPs extracts, however, have a complex physical–chemical composition and the recovery of target compounds requires several steps (i.e., coagulation and precipitation of impurities, adsorption on resins) based on the use of chemicals, solvents and high temperatures, which account for a significant part of the total production costs [6,7].

In this study, we used an extraction procedure for OPWPs that makes use of membrane technologies, micro-, nano- and ultrafiltration along with reverse osmosis as purification steps. The mixtures have no additional harmful components and a positive, albeit incomplete, environmental impact. We applied this technique to obtain the polyphenolic extracts from OPWPs with a distinct geographical origin across Italy, i.e., Apulia, Tuscany and Sicily. We previously reported that such extracts display a significant antioxidant activity, mostly due to HTyr in which they are prominently enriched [18,19]. Here, we assessed their abilities to affect cell viability both in normal and colorectal cancer (CRC)-derived human cell lines and disclosed an unprecedented mechanism underlying the anti-inflammatory activity.

## 2. Materials and Methods

### 2.1. Chemicals

All solvents, tyrosol and verbascoside used as reference standards were purchased from Sigma-Aldrich (Milan, Italy). Hydroxytyrosol (HTyr) was synthesized according to a procedure already described [20]. The Milli-Q-system (Millipore SA, Molsheim, France) was used to produce deionized water.

### 2.2. Sampling and OPWPs Extracts Methods

The three HTyr-enriched extracts were produced from different sites across Italy, namely Apulia, Tuscany and Sicily, henceforth named AE (Apulia Extract), TE (Tuscany Extract) and SE (Sicily Extract), respectively, as previously reported [14,18,19,21]. Briefly, AE was obtained from freeze-dried olive oil waste water from cultivars Coratina and Leccino (50:50) milling, produced in the Germinario’s farm, Molfetta (Apulia, Italy; 41°31′42.8″ N, 12°47′31.3″ E). TE was prepared from concentrated olive oil mill wastewater from cultivar Leccino and Frantoio, 50:50, sampled in Il Forbiciaio’s farm, from Piancastagnaio—Siena (Tuscany, Italy, 42°51′5″76 N, 11°41′9″24 E). SE was prepared from cultivar Leccino olive pomace, from Catania (Sicily, Italy, 37°29′32″ N, 15°4′13″ E). All extracts were stored in plastic vials at −20 °C until use.

Olive oil wastewaters AE and TE were obtained using previously described procedures [21]. For olive pomace extract, SE, to obtain a pulp, after olive oil production, the olives were pot-holed and acidified to pH = 2.5–4.0. The extract was obtained at room temperature using an aqueous solvent and an electrical pneumatic extractor. The ensuing solution was filtered by multiple steps (micro-, ultra- and nanofiltration). After reverse osmosis, the fraction was concentrated under vacuum by using a scraper evaporator series (C & G Depurazione Industriale Company, Florence, Italy) combined with a heat pump. All olive harvests were performed between November 2016 and January 2017.

### 2.3. Characterization of OPWPs Extracts

The extracts were characterized using high-performance liquid chromatography/diode array detector (HPLC/DAD) and the analytical profiles were compared with HTyr. The HPLC/DAD analyses were carried out using an HP 1200 liquid chromatograph (Agilent Technologies, Santa Clara, CA, USA), equipped with an analytical column (Lichrosorb RP18 250 × 4.60 mm i.d, 5 μm) (Merck, Darmstadt, Germany). The eluents were H_2_O adjusted to pH = 3.2 with HCOOH (solvent A) and CH_3_CN (solvent B). A four-step linear solvent gradient was used, starting from 100% of solvent A up to 100% of solvent B, for 88 min at a flow rate of 0.8 mL min^−1^ [14,18,19,21]. Analytical parameters were the following: precision 4.1%; accuracy: 96%; LOD: 0.33 mg Kg^−1^; LOQ: 1.1 mg Kg^−1^; range of concentration: 1 mg g^−1^–200 mg g^−1^. Phenolic compounds found in the extracts were identified by comparing retention times and UV/Vis spectra with those of the authentic standards. Each compound was characterized using a five-point regression curve built with the available standards.

### 2.4. Cell Culture and Treatments

All human cell lines used in this study were acquired from the American Type Culture Collection (ATCC, Rockville, MD, USA) and cultured at 37 °C under 5% CO_2_. The human normal colonic CCD-841CoN and WS1 fibroblast cell lines were cultured in DMEM (Life Technologies, Waltham, MA, USA), while human CRC-derived cell lines, HCT116 and LoVo, in RPMI 1640 (Life Technologies), were supplemented with 10% *v/v* fetal bovine serum (Life Technologies), 2 mM _L_-glutamine, 100 U mL^−1^ penicillin/streptomycin. oil production waste products (OPWPs) freeze-dried extracts were resuspended in sterile water at the concentration of 100 mg mL^−1^ (corresponding to their solubility). In all experiments, HCT116 cells were treated with 0.0154 mg mL^−1^ HTyr or 0.0154 mg mL^−1^ OPWPs extracts for 72 h; LoVo cells were treated with 0.0231 mg mL^−1^ HTyr or 0.0231 mg mL^−1^ OPWPs extracts for 72 h. To induce inflammation, 3 × 10^5^ HCT116 or LoVo cells were seeded in 6-well plates and incubated overnight at 37 °C in 5% CO_2_ atmosphere to enable cell adhesion. Cells were pretreated with HTyr or OPWPs for 72 h and then stimulated with 250 ng mL^−1^ lipopolysaccharide (LPS, L8274, Sigma-Aldrich) for the last 10 h. In some experiments both HCT116 and LoVo cells were treated with rosiglitazone (1 μM), a PPARγ selected agonist or with GW9662 (10 μM), a PPARγ irreversible antagonist, for 72 h (both from Cayman, Ann Arbor, MI, USA).

### 2.5. Viability Assay and Calculation of IC_50_ Value after the Treatment with HTyr

3 × 10^3^ cells/well CCD-841CoN or WS1, and 2 × 10^3^ cells/well HCT116 or LoVo cells were seeded into 96-well plates in complete medium and incubated overnight at 37 °C in 5% CO_2_ atmosphere to enable cell adhesion. Each analysis was carried out using cells thawed from three different stocks and seeded in the same plate. All cells were then exposed to increasing concentrations of HTyr as indicated: CCD-841CoN and WS1 at 50, 100, 150, 300, 600 and 900 µM for 72 h; HCT116 cells at 0, 25, 50, 100, 150, 200 and 300 µM for 72 h; LoVo cells at 0, 50, 100, 150, 200, 300 and 400 µM for 72 h. Cell density was assessed by the CyQuant™ Cell Viability Assay (Thermo Fisher Scientific, Waltham, MA, USA) according to the manufacturer’s instructions. Fluorescence was measured by the Tecan Infinite-Pro 200 plate-reader using excitation at 480 nm and emission at 535 nm. The calculation of IC_50_ value was performed with the support of GraphPad Prism software, version 8.1.1 (GraphPad software, Inc., San Diego, CA, USA). Data were transformed in log scale and normalized, setting the dose 0 µM as 100% and the maximum dose tested as 0%.

### 2.6. Flow Cytometry Analysis

3 × 10^5^ cells were seeded in 6-well plates, grown overnight and treated with HTyr or OPWPs extracts for 72 h. FITC Annexin V Apoptosis Detection Kit I (556547, Becton Dickinson—BD, San Jose, CA, USA) was used to stain cells and evaluate cell death by the BD FACS Verse flow cytometer, as previously reported [22]. BD Cycletest™ Plus DNA Reagent Kit (340242, Becton Dickinson—BD) was used for cell cycle analysis. Flow data were analyzed with the FACSuite 1.0.6 software (Becton Dickinson—BD).

### 2.7. Western Blot Analysis

Protein extracts were analyzed as previously reported [23]. Briefly, the cells were lysed by an ice-cold lysis buffer containing 25 mM Tris-HCl, pH 7.5, 150 mM NaCl, 2 mM EDTA, 1% TritonX-100, 1% sodium deoxycholate, 0.1% SDS, a cocktail of protease and phosphatase inhibitors (Roche, Basel, Switzerland). Thirty µg of total protein extracts were heated at 95 °C for 5 min and loaded on reducing polyacrylamide gels, transferred onto a PVDF membrane, subsequently blocked with 5% non-fat dry milk. Antibodies to PPARγ (sc-7273) were from Santa Cruz Biotechnology (Dallas, TX, USA); p-NF-κB (GTX50254) and NF-κB (GTX102090) from Genetex (Irvine, CA, USA); β-Actin (F-3022) from Sigma-Aldrich. Anti-mouse or anti-rabbit antibodies, conjugated with horseradish peroxidase, were used as secondary antibodies. Clarity western ECL Substrate (#1705061, BIO-RAD, Hercules, CA, USA) was used to detect bands, using ChemiDoc XRS (BIO-RAD). Bands’ intensity was analyzed by ImageLab software (BIO-RAD). Some blots were cut and probed with different antibodies for different proteins including β-Actin. In some cases, to examine proteins of similar molecular weight, the PVDF membranes were subjected to a mild stripping protocol as recommended by Abcam (Cambridge, UK).

### 2.8. RNA Extraction and Quantitative Real-Time PCR Analysis

Total RNA was extracted using TRIZOL^®^ Reagent (Invitrogen, Carlsbad, CA, USA) following manufacturer’s instructions. To synthesize the cDNA, 1 µg of total RNA was reverse transcribed by using SuperScript III Reverse Transcriptase (18080044, Thermo Fisher, Life Technologies). qRT-PCR was performed with PowerUp™ SYBR™ Green Master Mix (A25742 Applied Biosystems, Waltham, MA, USA) using the QuantStudio 5 apparatus (Applied Biosystems) and the amplification conditions suggested by the manufacturer. *18S* RNA was used as the housekeeping gene, as already reported [24]. The sequences of the used primers and the relative annealing temperatures are reported here:

*TNF-α* (accession number: NM_000594)
*TNF-α* FW GAACATCCAACCTTCCCAAACG
*TNF-α* RV GACCCTAAGCCCCCAATTCTC
T = 62 °C

*Il-8* (*CXCL8*; accession number: NM_001354840)
*Il-8* FW TTGGCAGCCTTCCTGATTTCT
*Il-8* RV TTTCCTTGGGGTCCAGACAGA
T = 58 °C

*CD36* (accession number: NM_001001548)
*CD36* FW GAGAACTGTTATGGGGCTAT
*CD36* RV TTCAACTGGAGAGGCAAAGG
T = 58 °C

*INSIG1* (accession number: NM_001346590)
*INSIG1* FW GACAGTCACCTCGGAGAACCCCAC
*INSIG1* RV ACCGTGACGCCTCCTGAGAAAAATA
T = 60 °C

*18S* (accession number: NR_146151.1)
*18S* FW GGGAGCCTGAGAAACGGC
*18S* RV GGGTCGGGAGTGGGTAATTT
T = 60 °C

Fold change is calculated with the formula: Fold change = 2^−ΔΔCT^

where −ΔΔC_T_ = − (ΔC_T,Goi_ − ΔC_T,Ref_), Goi = Gene of interest, Ref = Reference gene

### 2.9. Enzyme-Linked Immunosorbent Assay (ELISA)

HCT116 and LoVo cells were seeded in 6-well plates (3 × 10^5^ cells/well) and treated as described above. At the end of treatments, media were collected and IL-8 or TNF-α protein levels measured by using specific ELISA kits (KHC0081 and BMS223-4, respectively, Invitrogen); some samples were diluted for the assay and the absorbance value was multiplied by the corresponding dilution factor, following the manufacturer’s instructions.

### 2.10. Statistical Analysis

All data are shown as mean ± SD of at least two independent experiments. For HTyr treatments statistical significance was calculated using *t*-test and significance shown as # *p* ≤ 0.05, ## *p* ≤ 0.01, ### *p* ≤ 0.001. #### *p* ≤ 0.0001. For the treatments with OPWPs extracts, ANOVA with Dunnett’s post-test was performed and significance shown as * *p* ≤ 0.05, ** *p* ≤ 0.01, *** *p* ≤ 0.001. **** *p* ≤ 0.0001.

In the experiments where the LPS treated cells are compared to untreated cells, the statistical significance was calculated using *t*-test and shown as # *p* ≤ 0.05, ## *p* ≤ 0.01, ### *p* ≤ 0.001, #### *p* ≤ 0.0001. The ANOVA with Dunnett’s post-test was used, instead, to calculate the statistical significance in the experiments that compare cells exposed to HTyr or the OPWPs extracts in combination with LPS versus cells treated with LPS alone and shown as * *p* ≤ 0.05, ** *p* ≤ 0.01, *** *p* ≤ 0.001. **** *p* ≤ 0.0001. Statistical analysis was made using GraphPad Prism software, version 8.1.1 (GraphPad software, Inc.).

## 3. Results

### 3.1. OPWPs Extracts Characterization

**Figure 1:**
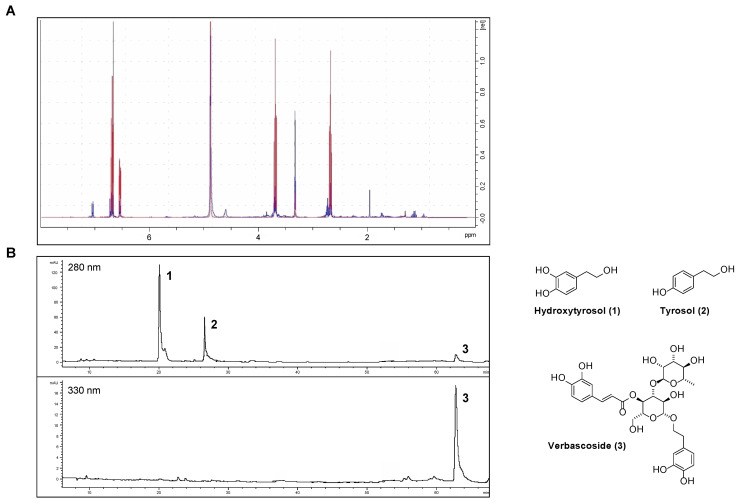
Chemical characterization of the Tuscany Extract (TE): (**A**) ^1^H NMR spectra of HTyr (red) and TE (blue); (**B**) HPLC chromatogram of TE a 280 and 330 nm.

Phenolics were extracted from OPWPs production lines processing different olive varieties obtained from three regions across Italy, namely Apulia, Tuscany and Sicily, and referred to as AE, TE and SE, respectively. AE and TE were obtained from olive oil waste waters while SE was from olive pomace; in all cases, the extraction involved a large-scale pilot process based on membrane technologies, thus being inherently ecofriendly and designed to obtain preferentially low molecular weight phenols, including HTyr. The filtration steps involved micro-, ultra- and nanofiltration down to a final step of reverse osmosis [14,18,19,21]. The extracts were analyzed by HPLC-DAD and NMR. In particular, we recently reported AE and SE characterization [18,19] while here we present the analytical determinations relative to TE. The Nuclear Magnetic Resonance spectra (^1^H NMR) of HTyr [20] and TE are shown in Figure 1A, while the HPLC chromatogram of TE is in Figure 1B. HPLC profile identifies and quantifies the amount of HTyr and other polyphenols found in TE (see Table 1, column 3); NMR spectra confirm the presence of HTyr in TE. In fact, the typical aromatic signals of HTyr at 6.53–6.70 ppm (red) are overlapping with those of TE (blue).

The content of the major components of the three extracts, as from HPLC analysis, is summarized in Table 1. Specifically, AE total phenol content was intermediate (50.74 mg g^−1^), with 63% of HTyr and the remaining percentage of tyrosol and verbascoside. SE showed the higher total phenolic content (149.74 mg g^−1^) about 77% of which was HTyr. The TE extract showed the lowest total phenol content (32.62 mg g^−1^), consisting of 36% HTyr, 46% tyrosol and, for the remaining percent, verbascoside. Notably, verbascoside was not found in SE. Considering only HTyr and tyrosol, they constitute 71.5%, 82.1% and 100% of the total phenolic compounds of AE, TE and SE, respectively.

### 3.2. Hydroxytyrosol and OPWPs Extracts Affect Cell Viability In Vitro

In order to evaluate the biological effects of the OPWPs extracts, we first assessed the viability of the treated cells by calculating the IC_50_ for HTyr. This is the most abundant phenolic component of the extracts, and, for this reason, used as a reference compound. IC_50_ is a measure of the concentration of a given molecule to inhibit a biological or biochemical function, in this case cell viability, by 50% [25].

As a cell model system in vitro, we selected the colonic epithelium as intestinal cells are one of the first stations where polyphenols introduced with the diet are absorbed and exert their biological activity. We chose HCT116 and LoVo cell lines, derived from human CRC, as they are widely employed as valid tester-cell types and allow the ruling out of effects that can be specific to a single cell line [26,27]. In addition, CRC is one of the human tumors with a very high incidence and subjected to many environmental cues such as lifestyle, exercise and dietary habits [28,29].

**Figure 2:**
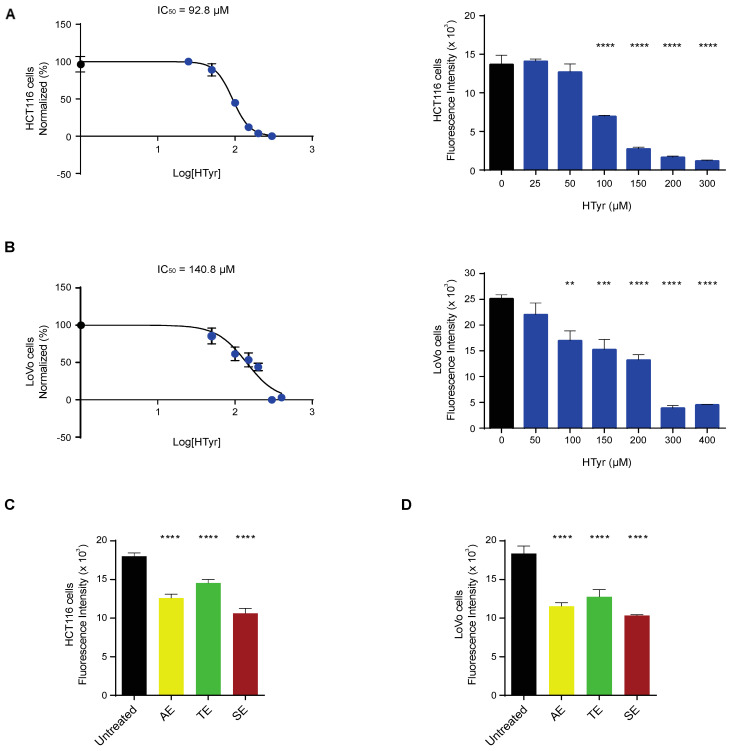
Hydroxytyrosol and OPWPs extracts influence colorectal cancer cells viability: (**A**) Cell viability of HCT116 cells treated with increasing concentrations of HTyr (from 0 to 300 µM) for 72 h assessed by fluorescence intensity and reported here transformed in a log scale or as histograms. (**B**) Cell viability of LoVo cells treated with increasing concentrations of HTyr (from 0 to 400 µM) for 72 h assessed by fluorescence intensity and reported here transformed in a log scale or as histograms. (**C**) Cell viability of HCT116 cells treated with 0.0154 mg mL^−1^ OPWPs extracts for 72 h assessed by fluorescence intensity and reported as histograms. (**D**) Cell viability of LoVo cells treated with 0.0231 mg mL^−1^ OPWPs extracts for 72 h assessed by fluorescence intensity and reported as histograms. Statistical significance is considered when ** *p* ≤ 0.01, *** *p* ≤ 0.001, **** *p* ≤ 0.0001 (ANOVA with Dunnett’s post-test).

We treated HCT116 and LoVo cells with increasing doses of HTyr for 72 h, as indicated in the materials and methods section, and evaluated cell viability by the CyQUANT proliferation assay. Cell viability was reduced in a dose-dependent manner, with an IC_50_ calculated at 92.83 µM for HCT116 and 140.8 µM for LoVo cells (Figure 2A,B). Experiments carried out for shorter times (24 and 48 h) produced similar but less intense results and for this reason are not reported in this study. We then treated HCT116 and LoVo cells with the OPWPs extracts at the concentrations of 0.0154 and 0.0231 mg mL^−1^ corresponding to the HTyr values of 100 µM and 150 µM, respectively, in order to compare the effects of a pure molecule (HTyr) with those obtained with a mixture containing equivalent amounts of total polyphenols. In HCT116 cells, the AE, TE and SE extracts reduced cell viability of 30%, 19% and 41%, respectively (Figure 2C), while in LoVo cells the reduction was of 36%, 30% and 43% for the AE, TE and SE, respectively (Figure 2D). These data rule out cytotoxic effects of OPWPs treatments carried out at the doses corresponding to HTyr IC_50_. Among the extracts, SE has the strongest inhibitory effect, about 40%, in both tumor cell lines, that can be entirely ascribed to HTyr, tyrosol and derivatives, present at the highest concentration (see Table 1). The AE and TE exerted a proportionally lower inhibitory effect, consistent with the lower content of HTyr, tyrosol and derivatives, about 24.2% and 17% of the SE, respectively.

**Figure 3:**
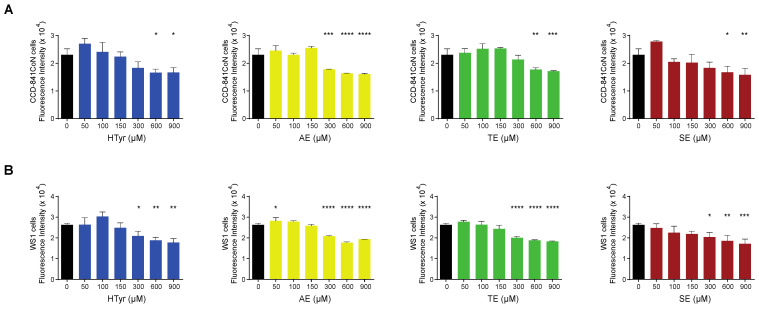
Normal cells viability exposed to hydroxytyrosol and OPWPs extracts: (**A**) Cell viability of CCD-841CoN normal colon cells exposed to increasing doses of HTyr or OPWPs extracts (from 0 to 900 μM) for 72 h assessed by fluorescence intensity and reported as histograms. (**B**) Cell viability of WS1 normal fibroblasts exposed to increasing doses of HTyr or OPWPs extracts (from 0 to 900 μM) for 72 h assessed by fluorescence intensity and reported as histograms. Statistical significance is considered when * *p* ≤ 0.05, ** *p* ≤ 0.01, *** *p* ≤ 0.001, **** *p* ≤ 0.0001 (ANOVA with Dunnett’s post-test).

We also assessed the effects of HTyr and the polyphenolic mixtures on normal cells and compared them with those obtained in tumor cells. To this goal, we selected a normal colonic (CCD-841CoN) and a normal skin fibroblast (WS1) cell line to rule out cell-type specific effects and have more common results considering their different embryonic origin. Preliminary experiments indicated that normal cells are less responsive than tumor cells; for this reason, they were exposed to a much wider range of HTyr or OPWPs doses (from 0 to 900 µM) for 72 h. Strikingly, in CCD-841CoN cells, viability started to be significantly reduced at the highest doses used (600 and 900 µM) (Figure 3A); in WS1, viability started to be reduced at 300 µM (Figure 3B). In both cases, cell viability diminished by about 20–35%, even at highest dose tested, suggesting that these compounds influence normal cells viability only at very high concentrations. On the basis of these results, we decided not to investigate the normal cell lines further in all subsequent experiments as they exhibit no effects at the doses employed in our experimental setting for the tumor cell lines.

### 3.3. Hydroxytyrosol and OPWPs Extracts Induce Apoptosis in Colorectal Cancer Cells

**Figure 4:**
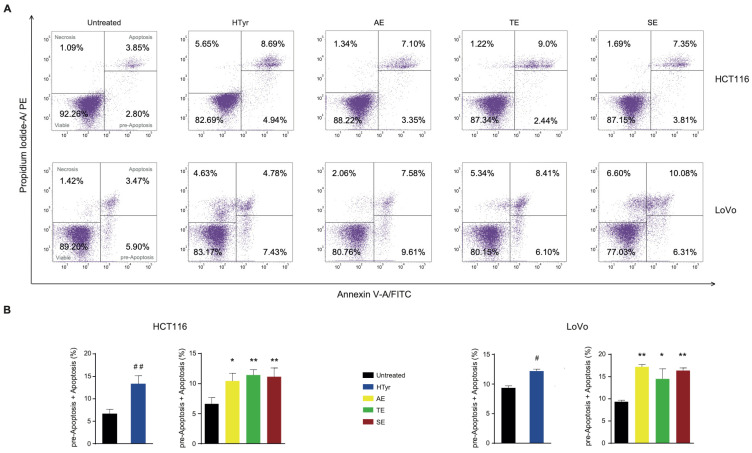
Hydroxytyrosol and OPWPs extracts promote programmed cell death in colorectal cancer cells: (**A**) Flow cytometry analysis of Annexin V/Propidium Iodide of HCT116 stained cells, treated with 0.0154 mg mL^−1^ of either HTyr or OPWPs extracts (upper panel) and LoVo cells treated with 0.0231 mg mL^−1^ of either HTyr or OPWPs extracts (lower panel) for 72 h. Viable cells are comprised in the lower left quadrant; necrotic cells in the upper left; pre-apoptotic cells in the lower right and apoptotic cells in the upper right. The number reported in each quadrant indicates the relative percentage of cells. (**B**) The histograms report the percentages of cells undergoing apoptosis calculated by adding both the pre-apoptotic and apoptotic cells treated with HTyr or OPWPs extracts with respect to untreated cells. Statistical significance is considered when # *p* ≤ 0.05, ## *p* ≤ 0.01 (*t*-test) or * *p* ≤ 0.05, ** *p* ≤ 0.01 (ANOVA with Dunnett’s post-test).

We wondered whether the reduced cell viability elicited by HTyr and OPWPs extracts was due to an increase in cell death. To this end, we stained the cells with propidium iodide (PI) and annexin V and assessed the amounts of cells undergoing apoptosis by flow cytometry. Apoptosis is a programmed cell death characterized by morphological features such as loss of membrane integrity, condensation of the cytoplasm and nucleus and inter-nucleosomal cleavage and degradation of DNA [30,31]. Treatment of HCT116 cells with HTyr for 72 h induced a 2-fold increase in apoptotic cells (13.63%) with respect to untreated cells (6.65%) (Figure 4), considering those in the early apoptotic stage as committed to die and those in the late apoptotic stage. The phenolic extracts stimulated apoptosis at a slightly lower level than HTyr (13.63% vs. AE 10.45%, TE 11.44% and SE 11.16%). Since the amount of HTyr in each extract is variable with highest content in the SE and the induction of apoptosis was similar among all extracts, we hypothesized that other components, as tyrosol and derivatives, can contribute to this effect.

In LoVo cells, HTyr produced only a 1.3-fold increase in apoptotic cells (12.21% vs. 9.37%). The OPWPs extracts induced a higher and variable percentage of apoptotic cells (AE 17.19%, TE 14.51% and SE 16.39%). This finding was remarkable because, although OPWPs have a content of HTyr lower than the control, the percentage of dead cells is higher, supporting that tyrosol and derivatives present in this mixture play a role. Altogether, the results obtained with both CRC-derived cell lines suggest that components of the extracts other than HTyr, specifically tyrosol and derivatives, are important factors in this process, highlighting possible scenarios of synergistic effects.

### 3.4. Hydroxytyrosol and OPWPs Extracts Cause a G_2_/M Cell Cycle Arrest in Colorectal Cancer Cells

To further explain the cell growth inhibition exerted by HTyr and OPWPs extracts, we performed a cell cycle test. Cell cycle is formed by well-defined phases: in the G_0_/G_1_ phase, cells get ready to replicate the DNA in the S phase. In the following G_2_ phase, cells synthesize and accumulate all components necessary for cell division during the M phase. Cell cycle is controlled by regulatory checkpoints located at the G_1_/S border to check that the replicative apparatus is properly set and no DNA damages are present; the G_2_/M border checkpoint ensures that sufficient amounts of all components have been synthesized for cell division to occur. If checkpoints are not cleared, the cell cycle is blocked [32,33]. All these processes are governed by different actors that involve a family of protein kinases, cyclin-dependent kinases (Cdks), regulated by the rise and fall of cyclins [34].

**Figure 5:**
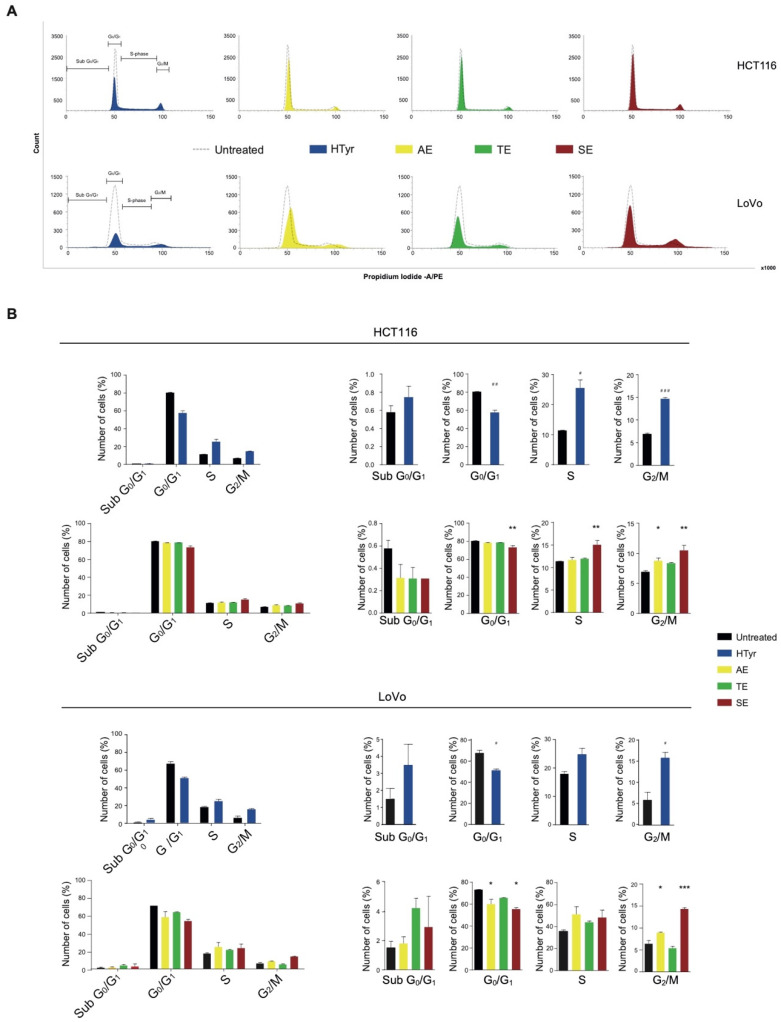
Hydroxytyrosol and OPWPs extracts induce a cell cycle arrest in colorectal cancer cells: (**A**) Cell cycle flow cytometry analysis of HCT116 cells treated with 0.0154 mg mL^−1^ of either HTyr or OPWPs extracts (upper panel) and LoVo cells treated with 0.0231 mg mL^−1^ of either HTyr or OPWPs extracts (lower panel) for 72 h. The horizontal bars indicate the various cell cycle phases in both cell lines. (**B**) The percentages of cells in each cell cycle phase for both cell lines were quantitated and reported as histograms considering all phases together (left) or showing details of each distinct phase (right). Statistical significance is considered when # *p* ≤ 0.05, ## *p* ≤ 0.01, ### *p* ≤ 0.001 (*t*-test) or * *p* ≤ 0.05, ** *p* ≤ 0.01, *** *p* ≤ 0.001 (ANOVA with Dunnett’s post-test).

Treatment of HCT116 cells with HTyr for 72 h caused a marked increase in cells in the G_2_/M phase (from 6.95 to 14.69%) and a consistent reduction of those in the G_0_/G_1_ phase (ranging from 80.52 to 57.70%) with respect to untreated cells, suggesting an induction of a G_2_/M cell cycle arrest (Figure 5). Furthermore, in LoVo cells, HTyr caused an increase in cells in the G_2_/M phase (from 5.88 to 15.77%) and a diminution of those in the G_0_/G_1_ phase (ranging from 66.97 to 50.69%). Administration of OPWPs extracts to both cell lines for 72 h at the concentrations reported above produced similar, albeit less intense, results. Specifically, AE and SE induced a G_2_/M cell cycle arrest in both cell lines, likely due to the different content of HTyr present in the mixtures.

Overall, the data shown so far suggest that HTyr and the OPWPs extracts inhibit CRC cells proliferation by inducing apoptosis mainly through tyrosol and derivatives, and by arresting the cell cycle mainly through HTyr.

### 3.5. Hydroxytyrosol and OPWPs Extracts Influence the Inflammatory Pathway in Colorectal Cancer Cells

**Figure 6:**
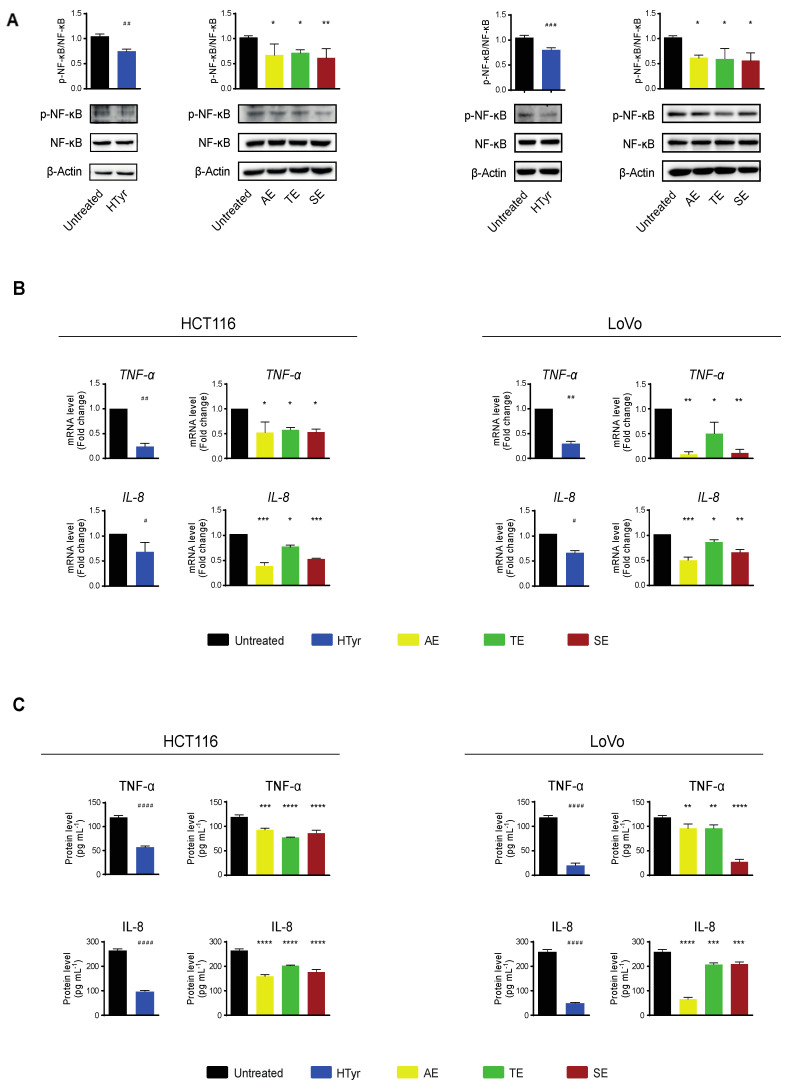
Hydroxytyrosol and the OPWPs extracts exhibit anti-inflammatory properties in colorectal cancer cells. (**A**) Western blot analysis of the phosphorylation levels of NF-κB normalized to total NF-κB in extracts from HCT116 and LoVo cells treated with HTyr or with the various extracts as indicated in the text for 72 h, with respect to untreated cells. β-Actin is shown as loading control. (**B**) qRT-PCR analysis of *TNF-α* and *IL-8* mRNAs in HCT116 and LoVo cells treated as in (**A**) and compared to untreated cells. (**C**) TNF-α and IL-8 protein levels evaluated in the culture medium of HCT116 and LoVo cells treated as in (**A**) with an ELISA assay. Statistical significance is considered when # *p* ≤ 0.05, ## *p* ≤ 0.01, ### *p* ≤ 0.001, #### *p* ≤ 0.0001 (*t*-test) or * *p* ≤ 0.05, ** *p* ≤ 0.01, *** *p* ≤ 0.001, **** *p* ≤ 0.0001 (ANOVA with Dunnett’s post-test).

We next investigated the molecular events underlying the cell growth inhibition by analyzing some of the intracellular signaling pathways involved. We focused on inflammation and immune response as these pathways have been reported to be affected by HTyr alone or by HTyr-containing extracts from olive leaves and extra virgin olive oil [35,36,37,38]. A critical role in this pathway is played by the nuclear factor κB (NF-κB) that, in resting cells, is sequestered in the cytosol through the binding to its inhibitor proteins IκBs (IκBα and IκBβ). In response to an inflammatory stimulus, IκB is phosphorylated, ubiquitinated and degraded by the proteasome. The NF-κB p65 subunit is phosphorylated, activated and, together with the partner p50, translocates to the nucleus where, as a hetero-dimeric transcription factor, it stimulates expression especially of inflammatory response genes [39,40]. We treated HCT116 and LoVo cells with HTyr at the above reported concentrations for 72 h and, in western blot analysis, found that NF-κB p65 phosphorylation was reduced by 30% in HCT116 and 25% in LoVo cells with respect to untreated cells. Interestingly, treatment with all OPWPs extracts produced a similar reduction in phosphorylation (Figure 6A). We next verified that the NF-κB downstream pathways were negatively regulated by analyzing the expression of some of the inflammatory response genes, such as cytokines and chemokines, that are transcriptionally controlled by this factor. The levels of *TNF-α* and *IL-8* mRNAs, as well as the amount of the cognate proteins secreted in the medium, were reduced upon treatment with HTyr in both cell lines, as by qRT-PCR and ELISA assays, respectively (Figure 6B,C). Treatment with OPWPs extracts induced the same pattern of repression, although less evident than HTyr alone. These results suggest that HTyr and OPWPs extracts restrain the inflammatory status already present in the tumor cell lines investigated.

**Figure 7:**
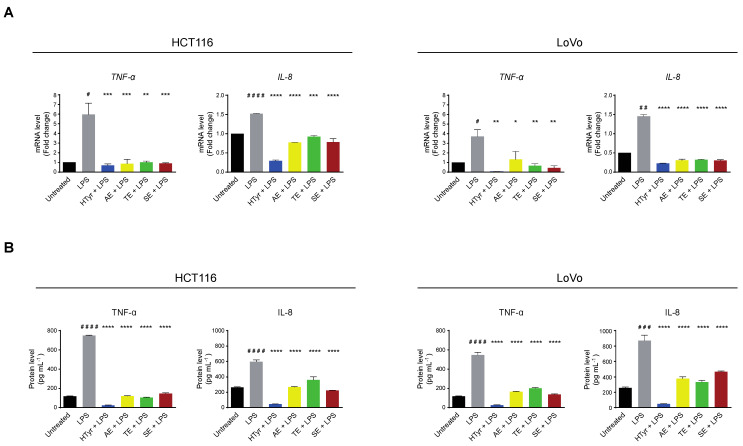
Hydroxytyrosol and OPWPs extracts prevent inflammation in HCT116 and LoVo cells. (**A**) qRT-PCR analysis of *TNF-α* and *IL-8* mRNAs in HCT116 (left) and LoVo cells (right) treated with HTyr or OPWPs extracts for 72 h and LPS for the last 10 h. (**B**) ELISA assay for TNF-α and IL-8 protein levels in the culture medium of HCT116 (left) and LoVo cells (right) treated as in (**A**). Statistical significance is considered when # *p* ≤ 0.05, ## *p* ≤ 0.01, ### *p* ≤ 0.001, #### *p* ≤ 0.0001 (*t*-test) or * *p* ≤ 0.05, ** *p* ≤ 0.01, *** *p* ≤ 0.001, **** *p* ≤ 0.0001 (ANOVA with Dunnett’s post-test).

We then verified whether the cells respond to an exogenous inflammatory stimulus, by treating them with the bacterial lipopolysaccharide (LPS) [250 ng mL^−1^], a known inflammatory inducer, for 10 h. In these experimental conditions, *TNF-α* and *IL-8* mRNAs and the corresponding secreted proteins, assessed as above, were upregulated indicating that the pathway was efficiently modulated by the applied challenge. Finally, we wondered if HTyr and OPWPs could counteract inflammation by pretreating the cells with HTyr or the extracts and, subsequently, with LPS. We chose this experimental approach as HTyr and the extracts require 72 h to display their effects, while LPS only requires 10 h, so that they are likely to be blunted in longer treatments. Interestingly, *TNF-α* and *IL-8* mRNAs and the corresponding secreted proteins were downregulated by HTyr to levels that were even lower than those of untreated cells. The OPWPs extracts reproduced the same pattern, albeit less intense (Figure 7).

Collectively, these results suggest that HTyr and the OPWPs extracts not only repress a basal inflammatory condition but also counteract any further increase caused by an additional proinflammatory challenge, exerting a preventive role.

### 3.6. Hydroxytyrosol and OPWPs Extracts Counteract Inflammation via PPARγ Activation

Finally, we attempted to clarify the mechanisms underlying the anti-inflammatory effects exerted by HTyr and OPWPs extracts. We focused on PPARγ, a nuclear receptor that plays regulatory roles in metabolism and cell differentiation but also in tissue inflammation and the immune system [41,42,43,44]. In melanoma cells, stimulation of PPARγ by its ligand pioglitazone influences the process triggered by Toll-like receptors (TLRs) with a reduction of the inflammatory state [45]. Furthermore, in macrophages, PPARγ was shown to negatively control the production of specific mediators [46].

**Figure 8:**
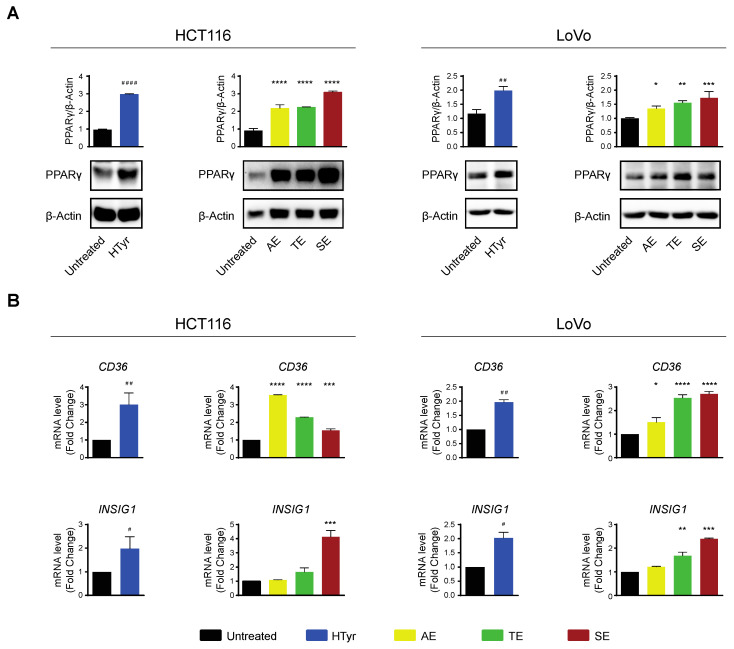
Hydroxytyrosol and the OPWPs extracts stimulate PPARγ and its target genes in colorectal cancer cells: (**A**) Western blot analysis of PPARγ in HCT116 and LoVo cells treated for 72 h with HTyr or the OPWPs extracts at the concentrations indicated in the text and compared to untreated cells. The histograms illustrate the ratio of PPARγ to β-Actin used for normalization. (**B**) Quantitative real time PCR analysis of *CD36* and *INSIG1* mRNAs in cells treated for 72 h with HTyr or the OPWPs extracts at the concentrations indicated in the text and compared to untreated cells. Statistical significance is considered when # *p* ≤ 0.05, ## *p* ≤ 0.01, #### *p* ≤ 0.0001 (*t*-test) or * *p* ≤ 0.05, ** *p* ≤ 0.01, *** *p* ≤ 0.001, **** *p* ≤ 0.0001 (ANOVA with Dunnett’s post-test).

**Figure 9:**
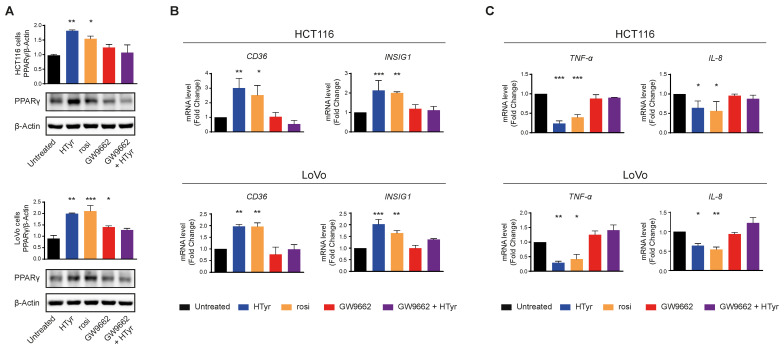
PPARγ mediates the anti-inflammatory effects of hydroxytyrosol and OPWPs extracts in colorectal cancer cells: (**A**) Western blot analysis of PPARγ in cells treated with HTyr (0.0154 mg mL^−1^ for HCT116 cells and 0.0231 mg mL^−1^ for LoVo cells), rosiglitazone (1 μM) or GW9662 (10 μM), or with HTyr and GW9662 in combination for 72 h and compared to untreated cells. The histograms illustrate the ratio of PPARγ to β-Actin used for normalization. (**B**) Quantitative real time PCR analysis of *CD36* and *INSIG1* mRNAs in both cell lines treated as in (**A**) and compared to untreated cells. (**C**) Quantitative real time PCR analysis of *TNF-α* and *IL-8* mRNAs in both cell lines treated as in (**A**) and compared to untreated cells. Statistical significance is considered when * *p* ≤ 0.05, ** *p* ≤ 0.01, *** *p* ≤ 0.001 (ANOVA with Dunnett’s post-test).

HCT116 and LoVo cells treated with HTyr or the phenolic extracts for 72 h, at the concentrations indicated above, displayed a marked increase in PPARγ with respect to untreated cells as shown by western blot (Figure 8A). We verified that the receptor was efficient in activating transcription of downstream genes by analyzing *CD36* and *INSIG1*, two well-known PPARγ target genes [47,48]. Their relative mRNAs increased upon treatment, as per qRT-PCR, confirming that the receptor was efficient in stimulating transcription (Figure 8B). We asked next whether this effect was carried out by HTyr acting as a specific PPARγ ligand; to this goal, we treated the cells with HTyr or rosiglitazone (1 µM), a well-known PPARγ agonist, for 72 h. As shown in Figure 9A, PPARγ levels increased indicating that both compounds bind the ligand binding pocket and stabilize the receptor. We assessed that PPARγ was transcriptionally active by estimating *CD36* and *INSIG1* mRNAs which, indeed, increased by approximately 2-fold with respect to untreated cells (Figure 9B). It has been shown that an active PPARγ is able to interfere with the expression of inflammatory genes through a mechanism called trans-repression [43]. To verify this, we found that *TNF-α* and *IL-8* mRNAs, representative of inflammatory cytokine genes, were reduced by HTyr and rosiglitazone, indicating that both compounds were similarly efficient in trans-repression. We then exposed the cells for the same time to GW9662 (10 μM), an irreversible PPARγ antagonist, and, by western blot analysis, found that it was able to stabilize the receptor, although, however, it was unable to transcriptionally activate target genes as *CD36* and *INSIG1* mRNAs remained unchanged with respect to untreated cells. Remarkably, also *TNF-α* and *IL-8* mRNAs remained stable with respect to basal levels, suggesting that PPARγ directly mediates trans-repression of inflammatory genes (Figure 9C). The combined treatment of HTyr and GW9662 for 72 h completely blunted the anti-inflammatory effect induced by HTyr, further supporting a PPARγ-dependent mode of action. Collectively, these last results indicate that HTyr likely acts as a PPARγ agonist, activates its transcriptional ability on target genes and interferes with the expression of inflammatory genes.

## 4. Discussion

The OPWPs represent a major environmental concern because they are produced at noticeable levels by even small-scale olive mills and contain large amounts of phenolic compounds responsible for a significant toxicity. These latter compounds, in fact, accumulate and permeate through the ground posing an alarming risk of contamination of the underlying aquifer and the resulting crops. For these reasons, a great deal of attention has been directed to the development of new technologies able to eliminate or reduce their toxicity down to acceptable levels. Among the many extractions procedures set-up and described in the literature, the one employed in this study combines advanced drying techniques with microfiltration, ultrafiltration and nanofiltration and reverse osmosis as purification steps. Another interesting characteristic of this procedure is that it can be scaled-up and used to dispose of large amounts of OPWPs with the goal to recover, on one hand, water that can be used for irrigation or other industrial purposes and, on the other, a phenolic mixture whose biological properties can be exploited as food additives for humans and animals alike or as constituents of cosmetic products [49,50].

In line with this reasoning, here we examined the biological properties of HTyr-enriched phenolic extracts obtained from three OPWPs produced in different regions of Italy by employing different production systems and experiencing different pedoclimatic conditions.

We selected the intestinal cells as a model system since they represent one of the first stations where the phenolic compounds or HTyr introduced with the diet are adsorbed and exert their effects. In addition, colonic cells can undergo malignant transformation and give rise to one of the most frequent tumors and the third cause of cancer-related death worldwide due to the high rate of metastasis [51]. CRC is still a public health and economic problem especially in developing countries where the incidence is still growing, although relevant achievements have been obtained in diagnosis and therapy. CRC onset/development can, in fact, be influenced by external stimuli, such as dietary habits, exercise and lifestyle [28,29].

We used two CRC-derived cell lines (HCT116 and LoVo) widely used as tester cells to rule out cell-specific effects and draw more general conclusions. To have a more comprehensive picture, we also tested a normal colonic (CCD-841CoN) and normal skin fibroblast cell line (SW1). The OPWPs were not toxic to tumor cells at the concentration corresponding to the IC_50_ value calculated for HTyr, with LoVo cells less responsive than HCT116 cells, as a third more HTyr was necessary to achieve the IC_50_. Given the different genetic, metabolic and epigenetic landscapes of the two cell lines, the results indicate that they respond to the treatments through a common mechanism. Reduced viability has been reported for another CRC-derived cell line, Caco-2, exposed to an OPWPs extract enriched mainly in non-extractable polyphenols and, thus, with a composition different from that reported here [52].

Remarkably, normal cells are less responsive in terms of viability to HTyr or OPWPs extracts than tumor cells, as 4/6-fold higher doses are required to obtain a reduction in viability of only 30%. The results suggest that these compounds, or mixtures thereof, affect normal cells only at the highest concentrations.

The reduced viability can be in part explained by the fact that cells undergo apoptosis or programmed cell death. In HCT116 cells, HTyr and OPWPs phenolic extracts increased the percentage of apoptotic cells by 2- and 1.5-fold, respectively, with respect to untreated cells, and with the same relative ratio between early and late apoptotic cells. Since all extracts elicited similar levels of apoptosis, despite a variable content of HTyr in each extract, we hypothesized that components of the extracts other than HTyr were contributing to this effect. In LoVo cells, HTyr induced a lower percentage of apoptosis (1.3-fold increase); notably, all the extracts induced apoptosis at a higher level than HTyr alone, providing support to our hypothesis that, in these cells as well, other components have a crucial role in stimulating this process.

Another possible reason for the observed reduction in cell viability is a cell cycle block. Many polyphenols, such as epigallocatechin gallate (EGCG) or black tea polyphenols, inhibit cell proliferation inducing a G_1_/S arrest [53,54]. Others, such as rosemary polyphenols, carnosol and carnosic acid result in a G_2_/M arrest [55]. HTyr has been reported to induce a cell cycle block at both sites [35,36,37,38]. Here, we report that HTyr blocks the cell cycle at G_2_/M in both cell lines and this effect is partially reproduced by the phenolic extracts [38,55]. Accordingly, the limited effects detected with the OPWPs extracts correlate with the lower content of HTyr.

Taken together, these results indicate that apoptosis is mainly promoted by the components of the extracts other than HTyr, which is, on the other hand, responsible for cell cycle arrest.

We report here that the OPWPs phenolic extracts exert anti-inflammatory activity, a characteristic previously investigated for olive oil or olive leaves extracts [49,50]. A single study showed that pretreatment of the OPWPs extracts with an immobilized tyrosinase resulted in enrichment of HTyr vs. tyrosol and inhibition of the inflammatory response in LPS-stimulated THP-1 monocytes [56].

Inflammation is the complex response of an organism to many harmful stimuli either toxic or of viral or bacterial origin and is associated with an active immune response. The major function of inflammation is to eliminate the etiologic agent, clear out necrotic cells and repair damaged tissues. This process usually resolves completely with the removal of the cause, healing of the tissue and recovery of the function; in some cases, for an impaired immune response or persistence of the inflammatory stimulus among others, the process persists giving rise to a chronic inflammatory status frequently associated with other pathologies. This condition and the presence of an unfavorable inflammatory microenvironment can foster tumor initiation/progression, as documented by a series of malignancies in which inflammatory markers are predominant [57]. A major role in this process is played by NF-κB that, in the active form, regulates the expression of target genes, many of which code for cytokines and chemokines involved in eliciting and/or maintaining inflammation. We show that HTyr and OPWPs extracts negatively regulate cellular inflammation, as documented by the reduced phosphorylation of NF-κB and, more importantly, by the reduced expression of some inflammatory target genes such as *TNF-α* and *IL-8* at both mRNA and protein levels. Interestingly, HTyr or OPWPs extracts also prevent inflammation. In fact, pretreating the cells with HTyr or OPWPs extracts restrains any further enhancement of inflammation, as that elicited by LPS, or even reducing it at levels below the basal ones. Our data, thus, support a dual anti-inflammatory activity of HTyr and the OPWPs extracts in tumor cells, in accordance with the reduced cell viability reported above, contributing to the overall anti-tumor effect of these compounds. More interestingly, HTyr and OPWPs extracts have basically no effects on normal cells at the doses used, suggesting that they may be employed in tumor treatments with the additional benefit to spare normal cells.

We also provide evidence that a major role in this process is played by PPARγ, a nuclear receptor involved in differentiation, lipid and glucose metabolism [41]. It has been shown that PPARγ negatively regulates the expression of proinflammatory genes by antagonizing the activities of other transcription factors such as members of the NF-κB family via a trans-repression mechanism [42,43,44]. Accordingly, the ligand-bound receptor undergoes an allosteric change that enables SUMOylation of a fraction of PPARγ that interacts with the corepressor complexes associated with NF-κB (p65/p50) [43]. As a consequence, the repressor complexes are no longer replaced on the promoters of inflammatory response genes, reducing their transcription and dampening the subsequent responses. Alternatively, PPARγ can directly interact with NF-κB reducing its transcriptional activity or can be recruited on the *IκB-α* promoter and stimulate its expression, ensuring a persistent level of the inhibitor and sequestration of NF-κB in the cytosol. Finally, PPARγ can regulate the IKK kinases activity that, in turn, modulates IκB-α stability or can compete for a limiting pool of coactivators [42,43,44,45,46].

The results reported here support that HTyr and the OPWPs extracts increase the amount of PPARγ that transcriptionally activates target genes such as *CD36* and *INSIG1*. At the same time, PPARγ reduces NF-κB phosphorylation, along with expression of target inflammatory genes as those coding for the crucial cytokines TNF-α and IL-8. Mechanistically, we show that this activity is mediated directly by PPARγ, as HTyr and rosiglitazone, a known PPARγ agonist, interact with its ligand binding pocket, stabilize the receptor and both activate its transcriptional potential to induce target genes and trans-repress proinflammatory genes. Interestingly, by using an irreversible PPARγ antagonist, GW9662, or administering a combination of HTyr and GW9662, both activation and trans-repression are impaired or completely blocked supporting a PPARγ-dependent mechanism.

These results suggest that HTyr and the extracts bind and activate PPARγ; we cannot rule out, however, that other secondary metabolites could contribute to the final anti-inflammatory effect through PPARγ activation. Poly-unsaturated fatty acids (PUFA) [58,59] and prostaglandins D_2_, in particular the PGJ_2_ derivatives [60,61], are known to play a role in the resolution of inflammation and thought of as possible PPARγ activators. Furthermore, specialized pro-resolving mediators (SPM), locally produced lipid mediators with a role in resolving inflammation, especially in macrophages or other immune cells, may also be induced and act in a PPARγ-independent manner [62,63]. Further studies are required to confirm the trans-repression process reported in this cell type and to fully clarify the underlying molecular basis. It is, in fact, possible that cell-specific mechanisms operate and different players, such as those mentioned above, participate in the process. Another intriguing question is to definitely establish whether HTyr acts as a PPARγ full or partial agonist. Full or partial agonists, in fact, can interact with different regions of the ligand binding pocket of the receptor ensuing the recruitment of diverse subsets of coactivators and hence stimulation of only a subset of target genes.

## 5. Conclusions

In this study, we extracted the polyphenols from the OPWPs derived from olive oil production by using a procedure that employs filtration and other separation steps to enrich the polyphenolic components of the waste. This technique is a step-forward as it reduces the levels of these polluting compounds in the environment according to the green and circular economy principles. In addition, it produces water that can be used for irrigation or other purposes and unmodified low molecular weight polyphenolic compounds. Furthermore, it likely opens new avenues for further research aimed at improving the extraction and enrichment methods to reduce the environmental impact.

We also showed that HTyr and polyphenolic extracts exhibit anti-inflammatory activity and prevent any further increase in CRC-derived cells. These characteristics add to the reduced cell viability, contributing to counteract the tumorigenic process. Remarkably, normal cells appear not to be affected at the employed doses, providing support for the use of such compounds as potential therapeutic agents in age and chronic inflammatory-related diseases, including cancers. The anti-inflammatory activity is mechanistically mediated by PPARγ likely through a trans-repression mechanism, that has not previously been disclosed in this cell model system.

## Figures and Tables

**Table 1:** Characterization of the OPWPs extracts by HPLC/DAD.

Compounds (mg g^−1^)	AE	TE	SE
HTyr	32.00	11.65	115.24
Tyr and derivatives	4.26	15.13	34.50
Verbascoside	14.48	5.84	---
Total polyphenols	50.74	32.62	149.74

The values reported are the average of three determinations with a standard error less than 2.5%.

## Data Availability

The data presented in this study are available in the article.

## Sample Availability

Samples of the compounds are available from the authors.
